# Dental replacement in Mesozoic birds: evidence from newly discovered Brazilian enantiornithines

**DOI:** 10.1038/s41598-021-98335-8

**Published:** 2021-09-30

**Authors:** Yun-Hsin Wu, Luis M. Chiappe, David J. Bottjer, William Nava, Agustín G. Martinelli

**Affiliations:** 1 Natural History Museum of Los Angeles County, Los Angeles, CA USA; 2 University of Southern California, Los Angeles, CA USA; 3 Museu de Paleontologia de Marília, Marília, São Paulo, Brazil; 4 Museo Argentino Ciencias Naturales “Bernardino Rivadavia”, Buenos Aires, Argentina

**Keywords:** Evolution, Evolutionary developmental biology, Palaeontology, Zoology

## Abstract

Polyphyodonty—multiple tooth generations—in Mesozoic birds has been confirmed since the nineteenth century. Their dental cycle had been assessed through sparse data from tooth roots revealed through broken jawbones and disattached teeth. However, detailed descriptions of their tooth cycling are lacking, and the specifics of their replacement patterns remain largely unknown. Here we present unprecedented µCT data from three enantiornithine specimens from the Upper Cretaceous of southeastern Brazil. The high resolution µCT data show an alternating dental replacement pattern in the premaxillae, consistent with the widespread pattern amongst extinct and extant reptiles. The dentary also reveals dental replacement at different stages. These results strongly suggest that an alternating pattern was typical of enantiornithine birds. µCT data show that new teeth start lingually within the alveoli, resorb roots of functional teeth and migrate labially into their pulp cavities at an early stage, similar to modern crocodilians. Our results imply that the control mechanism for tooth cycling is conserved during the transition between non-avian reptiles and birds. These first 3D reconstructions of enantiornithine dental replacement demonstrate that 3D data are essential to understand the evolution and deep homology of archosaurian tooth cycling.

## Introduction

Tooth replacement is a common phenomenon among vertebrates^1^. Unlike the diphyodonty of mammals, polyphyodonty (i.e., multiple generations of teeth)^2,3^ has been observed among fish^3,4^, amphibians^5^, and reptiles^6,7^. In particular, studies have clearly shown that reptiles have alternating patterns of tooth replacement, in which when the even-numbered teeth are under replacement, the odd-numbered teeth are not^1^. While teeth are replaced on a schedule that is independent of wear or damage^7^, the underlying regulatory mechanism of the replacement process has been difficult to understand. Within reptiles, modern studies of crocodilians—the only present-day toothed archosaurs—have demonstrated that the dental laminae within the alveoli (tooth sockets) form a niche of stem cells for the multiple generations of teeth, and that this mechanism is regulated by expression of a network of regulatory genes^8,9^. Even though our understanding of tooth development in extinct archosaurs, and its underlying regulatory mechanism, is still in its infancy, polyphyodonty has been documented for a range of extinct archosaurs^10–12^, including the toothed stem birds of the Mesozoic Era^13–15^.

The discovery of polyphyodonty and dental replacement in toothed stem birds dates back to the nineteenth century. Marsh^15^ reported replacement teeth inside resorption pits in the Late Cretaceous *Hesperornis* and *Ichthyornis*. In an attempt to understand the pattern of dental replacement of these birds, Edmund^1^ reviewed previous findings in avian dentition and concluded that the evidence was consistent with the presence of “replacement waves” in which stimuli moving in an anterior–posterior direction, generate waves of tooth replacement. Subsequently, based on the position of replacement teeth preserved within the roots of functional teeth, Martin et al.^14^ (see also Martin and Stewart^16^) concluded that the replacement teeth of *Archaeopteryx*, hesperornithiforms, and *Ichthyornis* developed vertically (albeit initially migrating from the lingual side) within the roots of functional teeth. Howgate^17^ confirmed the presence of an oval resorption pit on the root of a fully exposed tooth of the London specimen of *Archaeopteryx*. He also briefly mentioned that an alternating replacement pattern may have existed in the Berlin specimen but did not provide further description of this pattern. These early studies of avian dental replacement relied primarily on the external morphology of the fossils. However, we now know that tooth replacement and cycling are best understood through analysis of the jaws’ internal morphology, which requires X-ray imaging including computed tomography (CT) imaging among other methods.

**Figure 1:**
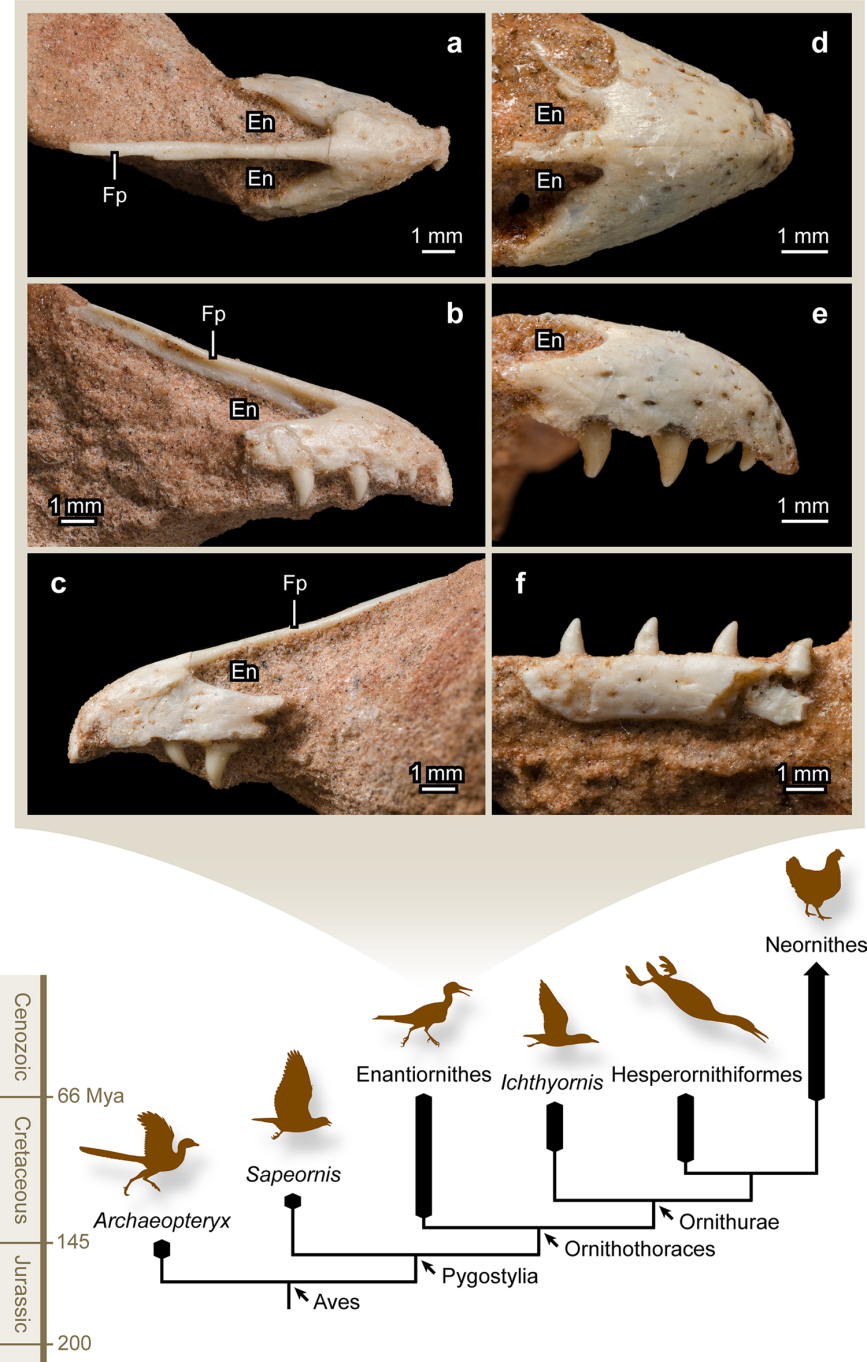
Photographs of the enantiornithine specimens MPM-90, MPM-373, and MPM-351, and a simplified cladogram highlighting the stem avian taxa discussed in this study. MPM-373: (**a**) dorsal view; (**b**) right lateral view; (**c**) left lateral view. MPM-90: (**d**) dorsal view; (**e**) right lateral view. MPM-351: (**f**) left lateral view. *En* external nares, *Fp* frontal process.

Such methods have been applied to more recent studies. A synchrotron scan of the jawbones of the Daiting specimen of *Archaeopteryx* revealed replacement teeth and traces of resorption on the roots of functional teeth, confirming an alternating tooth replacement pattern for this Late Jurassic bird^18^. Moreover, Dumont et al.^13^ utilized CT imaging techniques to make inferences on the implantation, attachment, and formation time of the teeth of *Hesperornis* and *Ichthyornis*. Based on these results, Dumont et al.^13^ suggested that in these stem birds the replacement teeth first formed at the lingual side of functional teeth, invading them through lingual resorption pits, and in the end expelling and replacing them. These authors provided important data on aspects of dental replacement in some of the most immediate toothed outgroups (the ornithurines *Hesperornis* and *Ichthyornis*) of living birds but the preservation of the scanned specimens limited the data available on the tooth cycling and replacement pattern throughout the jaw. Furthermore, to date no other study has examined these details in avian clades outside of *Archaeopteryx* and ornithurines (Fig. 1). In the present study, through micro-computed tomography (µCT) imaging, we examine three enantiornithine tooth-bearing bones from the Upper Cretaceous William’s Quarry, which was discovered in 2004 by one of us (WN) in the city of Presidente Prudente (São Paulo State) of southeastern Brazil, a locality of the Adamantina Formation (Bauru Group) containing hundreds of enantiornithine remains^19^. The exquisite preservation of these fossils allows examination of the emergence of replacement teeth within the jawbones and investigation of dental replacement of these stem birds in three dimensions throughout tooth-bearing bones. For the first time, these data allow testing specific hypotheses of dental replacement patterns in stem birds.

## Results

**Figure 2:**
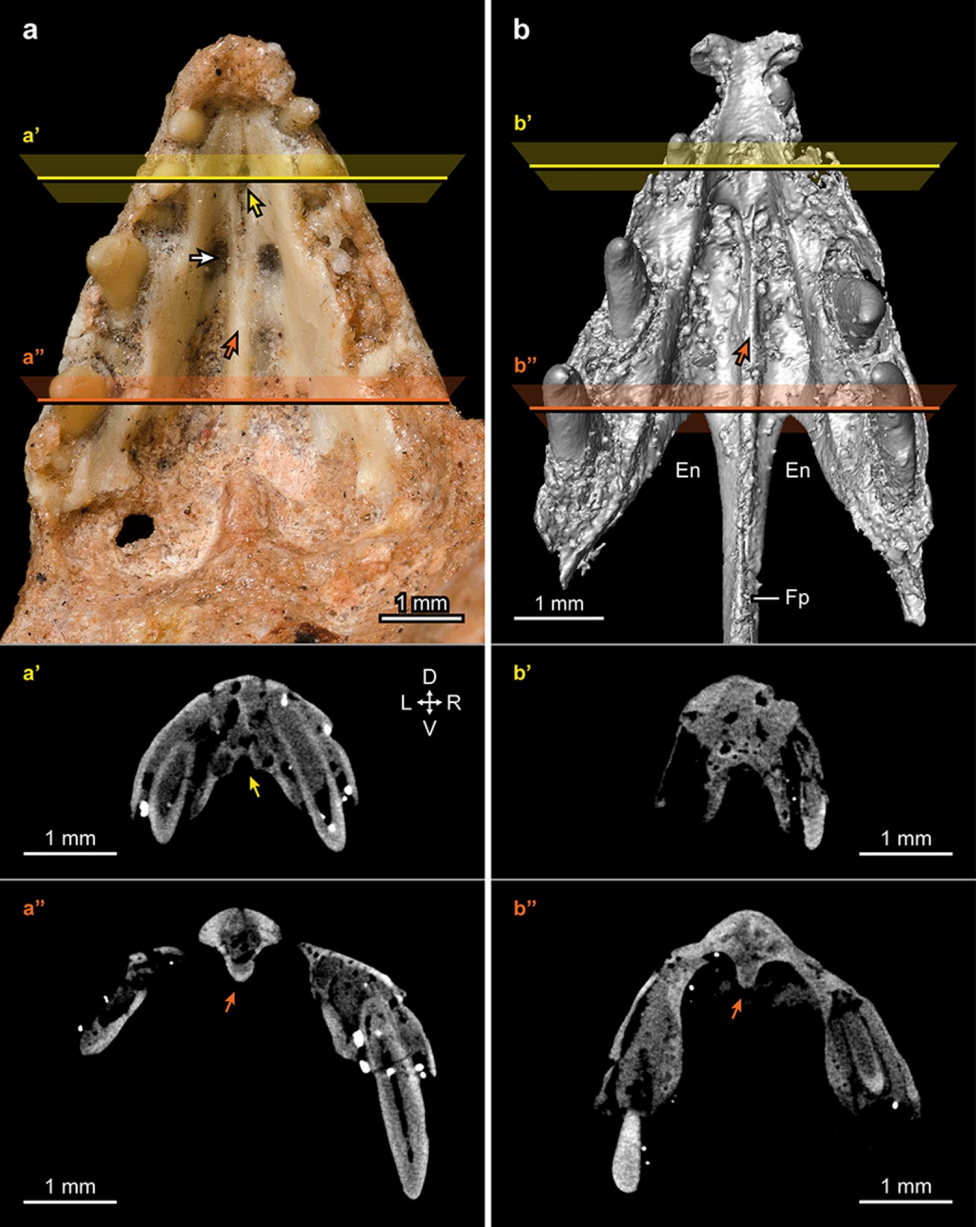
Ventral views of MPM-90 (**a**) and MPM-373 (**b**), and cross sections (**a′**, **a″**, **b′**, **b″**) of the premaxillae. Orientation of the cross sections is noted at the upper right corner of a’. *D* dorsal, *En* external nares, *Fp* frontal process, *L* left *R* right *V* ventral. Yellow arrows (**a**, **a′**) indicate the rostral palatal groove of MPM-90 (see text; this groove is not observed in the 3D reconstruction of MPM-373). Orange arrows (**a**, **a”**, **b**, **b”**) indicate the median palatal ridge developed in the posterior half of the pre-narial portion of the premaxillae (see text). The white arrow (**a**) indicates the small pits at the cranial end of the recesses, one on each side of the palatal ridge. MPM-373 (**b**) shown as a 3D reconstruction from the µCT scan.

The studied specimens consist of two sets of premaxillae (MPM-90 and MPM-373) and an incomplete left dentary (MPM-351) exquisitely preserved in three dimensions (Fig. 1). These specimens are housed at the Museu de Paleontologia de Marília (MPM), São Paulo State, Brazil. The two sets of premaxillae are very similar to one another, although MPM-373 is significantly smaller (prenarial length is 85.5% that of MPM-90. See Supplementary Table S1) and slightly more compressed laterally (Supplementary Fig. S1). The left and right premaxillary corpi are fused to one another in each of the specimens, although a faint line delineating the inter-premaxillary contact is visible in MPM-90 (Fig. 1d; Supplementary Fig. S1). While fusion of the premaxillary corpi is known for some enantiornithines (e.g., *Gobipteryx*^20^, *Longusunguis*^21^), such fusion is absent in many species within this group^22^. The premaxillae have four tooth positions, as is typical of enantiornithines and other toothed birds^23^, with most of their teeth—conical and slightly recurved—preserved in place. The general size of the erupted crowns is larger in the 3rd and 4th teeth than in the first two positions (Table 1; Fig. 1e). The smaller size of the first two premaxillary teeth is congruent with the morphology seen in a number of enantiornithines of different inferred lifestyles, including *Sulcavis*^24^, *Shenqiornis*^25^, and *Longipteryx*^26^. The frontal processes of the premaxillae, preserved in MPM-373, are long, slender, and fully fused with each other. These processes are typically unfused to one another in enantiornithines^20–23^, although their complete fusion was reported for the Early Cretaceous *Shangyang graciles*^22^. This process has approximately twice the length of the premaxillary corpus, as in the enantiornithines *Shangyang*, *Longusunguis*, and *Eoenantiornis*. The lateral sides of the frontal processes are recessed by a shallow, longitudinal groove (Fig. 1b; Supplementary Fig. S1). The angle formed between the longitudinal axes of the frontal and maxillary processes of MPM-373 is between 32 (right element) and 35 (left element) degrees (Supplementary Fig. S2), which indicates that the rostrum gently increased in depth caudally as in many enantiornithines including *Zhouornis*^27^, *Eoenantiornis*^28^, *Sulcavis*^24^, and *Pengornis*^29^. The lateral and dorsal surfaces of the premaxillary bodies of both specimens are scarred by mental foramina, although their distribution is not uniform, being less dense dorsally (Fig. 1a–e). Similar scarring of the premaxillary’s surface is known for a variety of enantiornithines^20,22,26–28^. The lateral dentigerous margin of MPM-90 is wavy but this is likely a preservational artifact as shown by the straight dentigerous margin of the left side of MPM-373 (Fig. 1c). Nonetheless, it is clear that the lateral (labial) edge of the dentigerous margin projected ventrally more than its medial (lingual) counterpart, thus leaving more of the crowns exposed lingually. In palatal view, only fully exposed in MPM-90, the left and right premaxillae are separated by a distinct, rostral groove that runs sagittally for almost half of the prenarial length of these bones (Fig. 2a–a′). Caudally, this groove gives way to a central ridge that is separated by parallel recesses on each side. The cranial ends of these recesses are further excavated by a small pit (Fig. 2a). This palatal ridge is also visible through µCT imaging in MPM-373 (Fig. 2b–b′). The overall morphology of the ventral premaxillae differs significantly from the shallow vaulted shape of this region in the enantiornithine *Gobipteryx*^20^.

**Table 1:** Total heights and erupted crown heights (mm) of teeth preserved in the three studied specimens.

Position	Functional	Replacement	Erupted crown height
**MPM-90 (premaxilla)**			
1(r)	1.85	0.14	0.54
2(r)	1.95	–	0.36
3(r)	3.02	–	1.18
4(r)	2.07	0.64	1.05
**MPM-373 (premaxilla)**			
2(r)	–	0.82	–
3(r)	2.52	–	1.08
4(r)	2.59	0.56	1.08
1(l)	–	0.63	–
3(l)	2.47	–	0.90
4(l)	2.40	0.66	1.16
**MPM-351 (dentary)**			
1(l)	2.18	0.41	1.10
2(l)	2.25	0.98	1.07
3(l)	2.37	–	1.05
4(l)	1.40+	–	1.03+

Total heights of both functional and replacement teeth are measured from 3D segmentation with Avizo Lite 9.2. Erupted crown heights are measured from photographs of the specimens following^48^.

Only the rostral portion of MPM-351 (left dentary) is preserved (Fig. 1f); this bone is broken at the level of its fourth alveolus, which is partially preserved and lacks evidence of a replacement tooth (it cannot be determined if a replacement tooth was absent or simply not preserved). The dorsal and ventral margins of this bone are parallel to one another, and its rostral end is slightly convex, angling caudally at approximately 43 degrees (Fig. 1f; Supplementary Fig. S2). The overall morphology of the dentary is remarkably similar to that of many enantiornithines including *Shenqiornis*^25^, *Zhouornis*^27^, and *Piscivorenantiornis*^30^. The lateral surface of the dentary is scarred by small, irregularly organized mental foramina. MPM-351 preserves the first four teeth, with the fourth somewhat broken. The dentary teeth are similar in shape and size, and evenly spaced. Like those in the premaxillae, these teeth are conical and slightly recurved, with heights ranging from 2.18 to 2.37 mm (Table 1).

Hundreds of partially articulated and isolated bird bones have been found at William’s Quarry, and all diagnosable ones can be assigned to enantiornithines^19^. This evidence suggests that the isolated cranial material described here also belongs to enantiornithines. This identification is further supported by the overall morphology of these specimens including the fact that functional teeth at the 1st and 2nd positions of the premaxillae are significantly smaller than those at the 3rd and 4th positions (Fig. 1e; Table 1), the long and slender frontal process of the premaxillae (Fig. 1a–b), the parallel nature of the dorsal and ventral margins of the dentary and angulation of its rostral end (Fig. 1f), and the clear absence of a mandibular symphysis (as evidenced by the morphology of MPM 351) (Fig. 1f). While some of these traits are also known for non-enantiornithine toothed birds (e.g., absence of mandibular symphysis in *Archaeopteryx*^31^, subparallel dorsal and ventral margin of dentary in hesperornithiforms^15^), their combined presence strongly supports the proposed identification of these specimens. Furthermore, as the record of Late Cretaceous birds is limited to enantiornithines and ornithuromorphs (including modern birds), the assignation of this material as belonging to Enantiornithes is congruent with the fact that the premaxilla of all toothed Late Cretaceous ornithuromorphs (e.g., *Ichthyornis*^32^, hesperornithiforms^33^) is devoid of teeth. The overall morphological similarity between the two sets of premaxillae—notwithstanding their size differences—suggest that these two specimens are likely to belong to the same species or to very closely related taxa.

**Figure 3:**
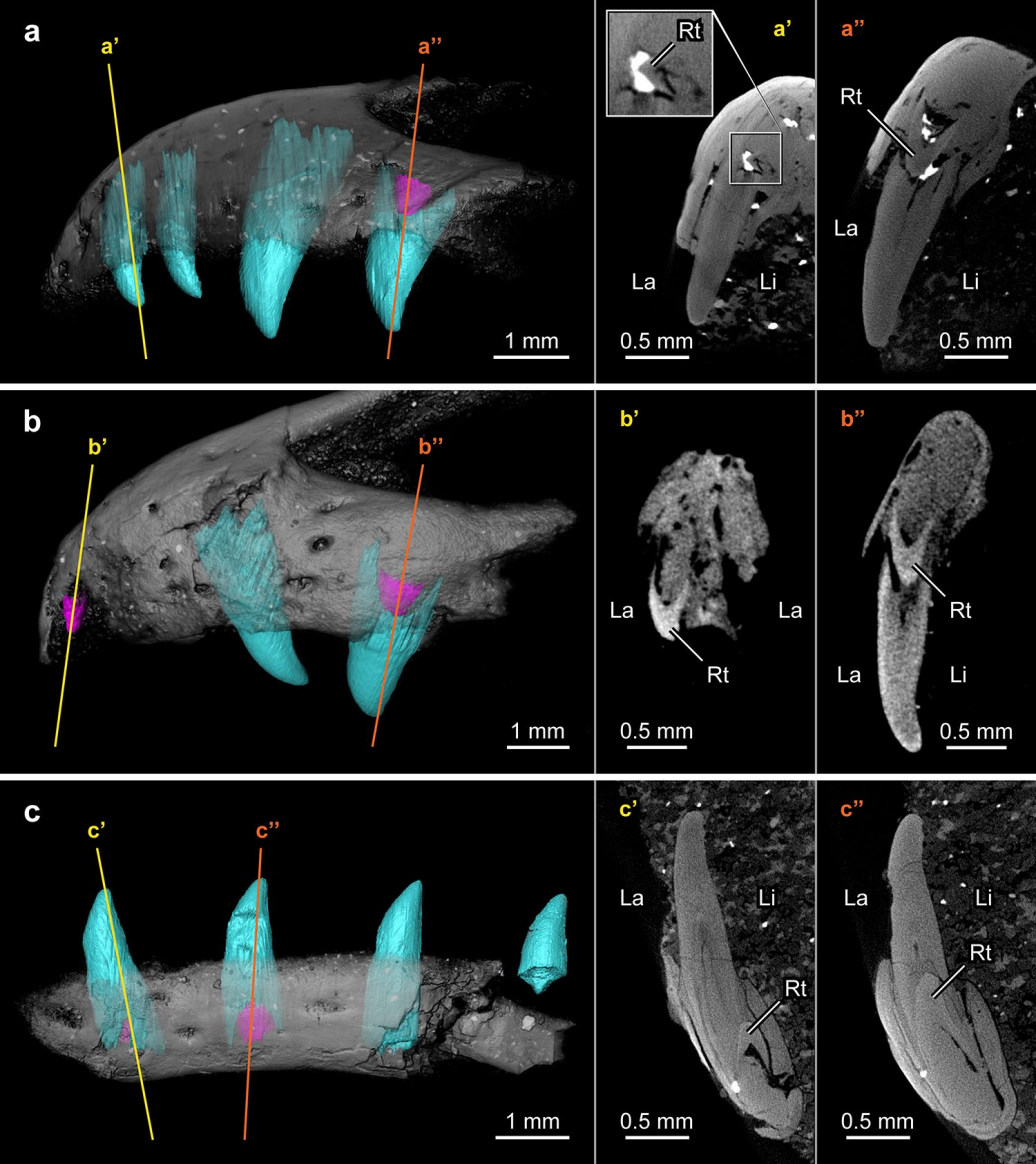
Dentition of MPM-90, MPM-373, and MPM-351 visualized through µCT imaging. (**a**–**c**) Labial views; (**a′**, **a″**, **b′**, **b″**, **c′**, **c”**) cross-sections of replacement teeth at the 1st (**a′**–**c′**), 2nd (**c″**) and 4th (**a″**, **b″**) positions. This figure was created to demonstrate the tooth distribution within jawbones and the resorption between the functional and replacement teeth. (**a**) Right lateral view of MPM-90, digitally flipped for comparison. (**b**) Left lateral view of MPM-373. (**c**) Left lateral view of MPM-351. Functional teeth in cyan; replacement teeth in magenta. Note that the functional teeth generally have more resorption on their lingual sides, except at the 4th tooth position of the right premaxilla of MPM-90 (**a″**). *Rt* replacement tooth, *La* labial, *Li* lingual.

**Figure 4:**
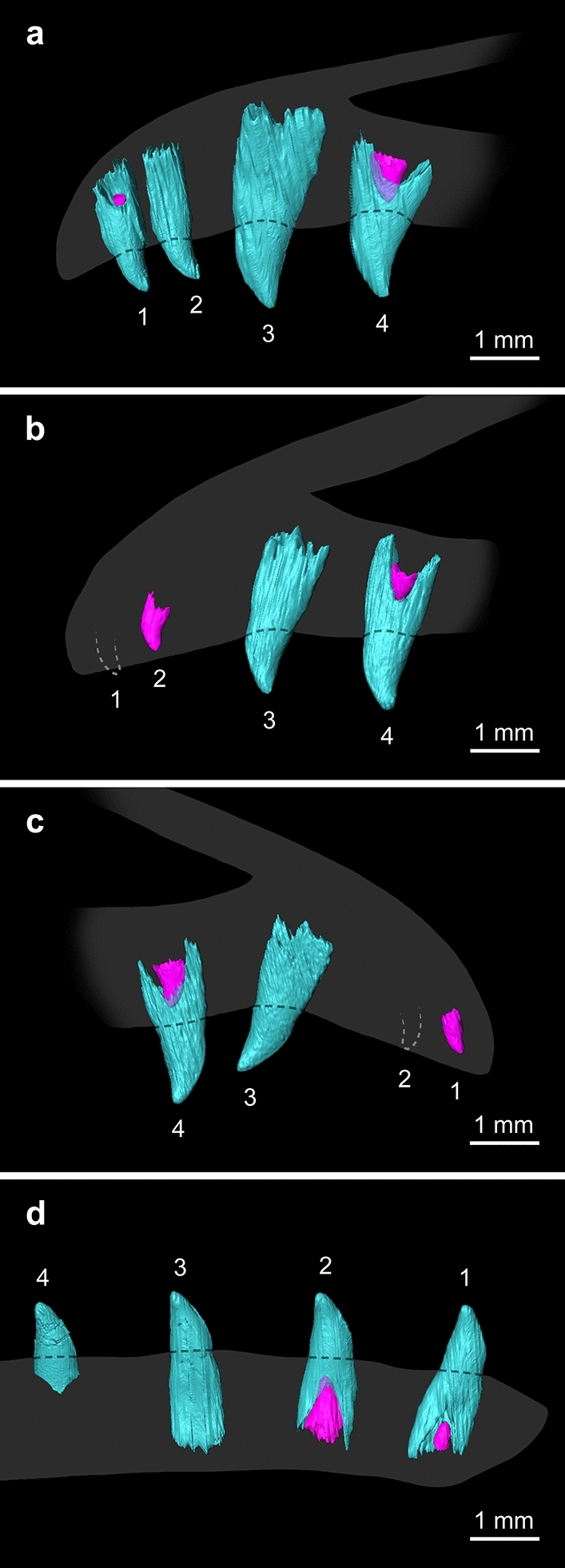
Preserved tooth rows and families of MPM-90, MPM-373, and MPM-351 visualized through uCT imaging. (**a**) Right lingual view of MPM-90. (**b**) Right lingual view of MPM-373. (**c**) Left lingual view of MPM-373. (**d**) Lingual view of MPM-351. Functional teeth in cyan; replacement teeth in magenta. White dashed lines (**b**, **c**) mark the positions of two missing teeth in MPM-373. Numbers denote tooth positions from mesial to distal. Silhouettes in gray provide schematic outlines of the tooth-bearing bones.

µCT scans of premaxilla MPM-90 show replacement teeth forming at the right 1st and 4th tooth positions (Fig. 3a–a″); the 2nd and 3rd tooth positions of the right element have no signs of root resorption or replacement tooth formation. Functional teeth are preserved in situ on the left side of MPM-90 at the first two positions. The lack of resorption on these functional teeth and the absence of replacement teeth visible within their alveoli, indicates that these positions were not being replaced at the time of death. µCT scans reveal that all teeth in MPM-90 have deep roots set in deep alveoli (Figs. 3a, 4a), even in the 1st and 4th functional teeth of the right element despite the resorption resulting from the formation of their replacement teeth. The replacement tooth forming in the lingual side of the right 1st position appears to be at its early stage because it is still small compared to the size of the functional tooth (Figs. 3a′, 4a). This replacement tooth may have been slightly displaced during postmortem, as the tooth is positioned lingual-labially with the apex pointing towards the labial side of the alveolus (Fig. 3a’). The root of the functional tooth at the 1st position exhibits significant root resorption (i.e., half of the height of the functional tooth) only on the lingual side (Fig. 3a′). At the right 4th position of MPM-90, the replacement tooth has grown to about one third of the height of the functional tooth (Table 1). The root of the later is resorbed significantly on both sides but more labially than lingually (Fig. 3a″), a condition rare in reptiles because of the lingually positioned dental lamina and lingual tooth replacement^6,7,34^. This rare resorption pattern may indicate either a labial replacement (labial to lingual; contra^34^) or most likely, an unusual odontoclast behavior.

MPM-373’s right premaxilla bears exposed teeth at the 2nd, 3rd, and 4th positions (Fig. 1b); µCT imaging reveals that the 1st position lacks a tooth. The one exposed at the 2nd position is a replacement tooth, which tip is exposed only because the dentigerous margin is incompletely preserved (Fig. 4b); those at the 3rd and 4th positions correspond to functional teeth. The left premaxilla of MPM-373 preserves a replacement tooth at the 1st position (Figs. 3b, 4c). µCT imaging shows that there are no teeth preserved at the left’s 2nd position. The teeth at the 3rd and 4th positions of the left premaxilla are functional teeth. Replacement teeth of similar sizes are also observed at the 4th positions (left and right) through µCT images, which also reveal the absence of replacement teeth at the 3rd positions, indicating symmetrical tooth cycles in at least these two positions (Fig. 4b–c). As in MPM-90, the functional teeth at the 3rd and 4th positions possess large roots set deep into the premaxilla. The functional teeth at the 4th positions of MPM-373 are more resorbed on the lingual side, showing typical reptilian lingual resorption that leads to a lingual to labial migration of the replacement tooth (Fig. 3b″). The replacement tooth at the 1st position and the functional tooth at the 3rd on the left side show evidence of possible dislocation. The former is preserved with its curvature facing forward; the latter is tilted some 30° (Fig. 4c).

µCT images of dentary MPM-351 show replacement teeth forming in the 1st and 2nd positions (Figs. 3c–c″, 4d). The replacement tooth at the 1st position is significantly smaller than that at the 2nd position. Considering the similar size of the functional teeth, the replacement tooth in the 1st position is interpreted as being at an earlier stage of development than its counterpart in the 2nd position. The functional tooth at the 3rd position has a deep root with no sign of resorption and no replacement tooth is visible within the alveolus (Fig. 4d). The fact that the replacement tooth of the 2nd position is at a later stage of development than those of the 1st and 3rd positions suggests an alternating pattern of dental replacement for MPM-351. However, this interpretation cannot be corroborated without the dental developmental stage of the 4th position, which is uncertain.

## Discussion

Our observations strongly suggest the presence of an alternating pattern of dental replacement for these enantiornithines. Comparative studies of such patterns often use a relation of replacement stages known as Zahnreihen, which consists of anterior to posterior diagonal tooth rows consisting of neighboring tooth families at sequential developmental stages^1,7,35^. When the distance in tooth position between these diagonal tooth rows (i.e., z-spacing) equals 2.0, it results in an alternating tooth replacement pattern in which all the odd-numbered teeth are under replacement and synchronized at the same development stages while the even-numbered teeth remain intact. However, if the z-spacing deviates from 2.0, the tooth rows may still present an alternating tooth replacement pattern, but one in which different tooth positions are under different stages of their tooth cycle^12,36^. Because the specimens studied here only have three to four tooth positions preserved in each dentigerous bone, it is difficult to estimate an average z-spacing. Nevertheless, the tooth rows of these enantiornithine jawbones appear to present alternating replacement patterns with different z-spacing that deviate from 2.0. While the 1st and the 2nd functional teeth of MPM-373 are not preserved, we would expect these teeth to have been smaller than the 3rd and 4th functional teeth (this is based on MPM-90 and other enantiornithines; see “Results”). Given this expected size difference between these tooth positions, the slightly larger replacement tooth at the 2nd position than that at the 4th position of the right premaxilla (Table 1) indicates that these teeth were at a late and relatively early developmental stage, respectively. Likewise, MPM-373’s left premaxilla has replacement teeth erupting at a similar height at the 1st and 4th tooth positions (Figs. 3b′–b″, 4c; Table 1), once again indicating a relatively late growth stage for the anterior position versus the posterior position. The teeth at the right 1st position and the left 2nd position are missing, but if MPM-373 were to have a symmetrical tooth cycle (as suggested by the condition in the 3rd and 4th positions), the late stages of replacement teeth of the anterior tooth positions would suggest a z-spacing smaller than 2.0, with an anterior to posterior tooth replacement^12,36^. In MPM-90’s right premaxilla, two erupting replacement teeth are observed at very early and late developmental stages in the 1st and the 4th tooth positions, respectively (Figs. 3a′–a″, 4a; Table 1). Dentary MPM-351 also has replacement teeth at different stages in the 1st and 2nd tooth positions (Figs. 3c′–c″, 4d; Table 1). MPM-90 has two consecutive positions with intact functional teeth, and MPM-351 has two consecutive positions with replacement teeth. They all have neighboring tooth positions undergoing different stages in the tooth cycle, which can be the result of alternating tooth replacement with z-spacing larger or smaller than two.

**Figure 5:**
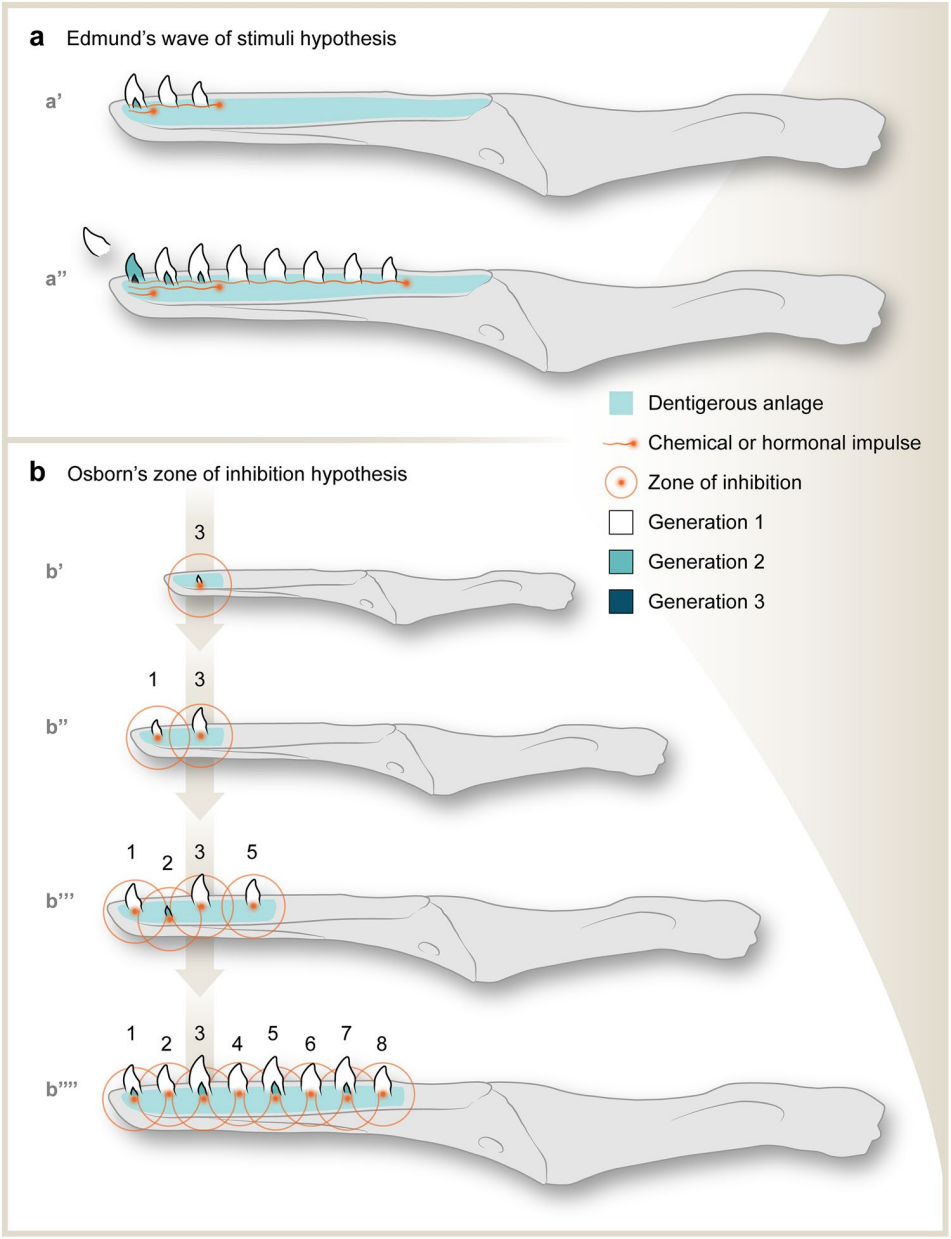
Schematic dentary of an enantiornithine contrasting Edmund’s wave of stimuli hypothesis (**a**) vs. Osborn’s zone of inhibition hypothesis (**b**) for control mechanisms of reptilian tooth replacement patterns. Modified from^7^. In Edmund’s hypothesis^1,11^, waves of chemical or hormonal stimulus pass caudally. When the first wave travels through the 3rd tooth position, it induces the development of the first three teeth (**a′**). As the second wave reaches the 1st tooth position (**a′**), a new tooth initiates from the dentigerous anlage (a foundation for cell proliferation and tooth development). The waves continue to travel caudally, and a new wave initiates a new generation of tooth formation (**a″**). In Osborn’s hypothesis^37,38^, when the first tooth develops, it generates an inhibition signal and suppresses the tooth formation in the neighboring area of anlage (**b″**). As the jaw and the anlage grow bigger, the distance between the teeth increases, and hence increases the space between the “zone of inhibition”, allowing new teeth to form from the anlage outside of the zone of inhibition (**b″**–**b′′′**). As the jaw and space between the teeth continue to expand craniocaudally, new teeth initiate in the neighboring tooth positions of the emerged teeth (**b′′′′**), and thus forming an alternating pattern. Numbers in (**b**) denote tooth positions from mesial to distal.

Mechanisms for reptilian tooth replacement patterns have been variously interpreted over the years. Edmund^1,11^ regarded this pattern as “waves” of teeth arising and being replaced, a mechanism he interpreted as triggered by impulses originating from the anterior end of the jaws and traveling posteriorly (Fig. 5a). Osborn^37,38^ proposed a different mechanistic hypothesis suggesting that developing teeth release inhibition signals into the surrounding mesenchyme and create zones of inhibition for the initiation of other teeth (Fig. 5b). These two hypotheses offer plausible explanations for the formation of alternating tooth replacement patterns, but further examination of the molecular basis for tooth development is necessary to understand the underlying control of these patterns. Molecular studies over the last decade have demonstrated that a suite of regulatory pathways may be candidates for either the triggering impulses in Edmund’s hypothesis or the inhibition signals in Osborn’s hypothesis. For example, the Wnt/β-catenin pathway has been shown to be essential for both tooth initiation and replacement^7,39^, and studies have documented the role of the related proteins in the maintenance of stem cells. Additional studies have shown that Wnt/β-catenin is inhibited by the Wnt antagonist, secreted frizzled-related protein (sfrp2), which has been found to express around (but not within) teeth in pythons and lizards^7^. Similarly, other secreted frizzle-related proteins (sfrp1) have been found to be present in the dental lamina bulge of alligator, while absent during the growth and development of new teeth^8^. Therefore, tooth formation and cycling in alligators is associated with the disappearance of sfrp1 and the activation of β-catenin in the stem cell niche^8^. Molecular studies of the controlling mechanisms underlying the pattern of tooth cycling in living reptiles have the potential for testing both Edmund’s and Osborn’s hypotheses, in turn clarifying key aspects of the development of the alternating tooth replacement pattern inferred from fossils of stem birds.

Teeth in these three enantiornithine specimens are all forming within the tooth sockets separated by porous interdental bones (Supplementary Fig. S3). The tooth replacement observed in these specimens indicates a labial migration of the new forming teeth. The position of the replacement teeth within the alveoli demonstrates that dental laminae were positioned lingually. Even if these three specimens were to belong to different enantiornithine taxa (albeit likely closely related), the first tooth families of MPM-351 and MPM-90 both show that the new forming replacement teeth started to resorb the root of the functional teeth and moved into the space occupied by the functional teeth at their early developmental stage. Martin et al*.*^14,16^ described the replacement teeth of A*rchaeopteryx*, and the hesperornithiforms *Hesperornis* and *Parahesperornis*, as developing in circular to oval resorption pits in the lingual side of their predecessors. They also suggested that the crown of the replacement tooth may have tilted labially when it entered the root of its predecessor, thus inferring a labial migration similar to their observations in crocodilians^16^. By comparing this phenomenon of tooth replacement in birds and crocodilians, Martin et al*.*^14,16^ argued that their similarity in dental replacement underscored a closer phylogenetic relationship (contra hypotheses supporting a dinosaurian origin of birds). Martin et al*.*’s description of the replacement teeth tilting labially implies that the initiation of replacement teeth starts lingual to the root of the functional teeth in the taxa studied by these authors. Our µCT-scan data show that in the Brazilian enantiornithines analyzed in the present study, all replacement teeth preserved at very early stages resorbed the lingual side of the functional teeth’s roots (Fig. 3a', c'); this indicates that the initiation of tooth formation occurred lingual to the functional teeth, thus confirming Martin et al*.*’s observations. Our observations indicate that during early development, the replacement teeth migrate into the pulp cavity of their functional teeth, subsequently growing within the space formerly occupied by the functional teeth. The replacement teeth at the 1st and 2nd tooth positions of MPM-351 do look like they are forming within the pulp cavity. However, while remaining right underneath the functional teeth, the 4th tooth positions at MPM-373 and MPM-90 already have significant resorption on the labial side of the functional teeth when the replacement teeth are only about one-fifth to one-third of the height of the functional teeth (Fig. 3a'', b''). Our data also show that the early labial migration in enantiornithine birds differs from the known pattern of dental replacement in non-avian theropods; for example, the replacement teeth of *Gorgosaurus*, *Allosaurus*, and *Coelophysis* resorbed into the lingual walls of their alveoli and remained parallel in coronal view without occupying the pulp cavity of the functional teeth until later in their development^34,40^. This shows that the process of tooth replacement in enantiornithines is more similar to that in crocodilians than to that in non-avian theropods, even if data from the latter derives from taxa that are not very close to the origin of birds. Future studies of non-avian theropods closer to the divergence of birds (e.g., non-avian paravians) may elucidate the evolution of the dental replacement pattern of stem birds, but given the voluminous evidence in support of the theropod origin of birds, the similarities between crocodilians and toothed birds are likely homoplastic (contra Martin et al*.*^14,16^).

Erickson^41^ proposed to assess tooth replacement rates by counting the difference between the incremental lines of von Ebner of a functional tooth and those of its subsequent replacement tooth of the same tooth family. This method has since been widely used to study the replacement rates and frequencies in multiple dinosaur groups^10,42^. Other than this measurement of replacement rates, studies have tried to infer general relative rates of replacement through the proportion of all teeth undergoing replacement at a given time^43^. The number of successional generations in a tooth family has also been considered as replacement frequency^44–46^. Based on their high-resolution synchrotron scans, Dumont et al*.*^13^ only observed a maximum of one replacement tooth forming lingual to the functional teeth of *Hesperornis* and *Ichthyornis*. Because other non-avian archosaurs had been observed as having multiple generations of replacement teeth in their tooth families (e.g.,^10,36^), Dumont et al*.* suggested that these toothed birds may have had oligophyodonty (i.e., reduced frequency of tooth replacement). Our observation of the three Brazilian enantiornithine specimens is consistent with those of Dumont et al*.*’s. All the tooth positions undergoing tooth replacement in MPM-90, MPM-373, and MPM-351 have only one replacement tooth in each alveolus. Hence, our data suggests that these enantiornithines only had a maximum of two generations (one functional and one replacement tooth) for each tooth family at a time. Although these birds share a lower number of dental generations at a given time when compared to their archosaurian relatives, it is unclear whether they also had a lower rate of replacement (i.e., frequency). Based on dentine incremental lines revealed by synchrotron scans, if those lines represent daily records, tooth formation duration in *Hesperornis* was estimated to be 66 days^13^, with the onset between two tooth generations being shorter. Nevertheless, the onset between two tooth generations and the number of tooth generations present at a time may also vary due to the growth of the skull through ontogenetic stages^46^. Histological and ontogenetic data of toothed birds will provide additional insights to the evolution of replacement rates and frequency, but unfortunately growth series of these birds are exceedingly rare.

The µCT-scans of MPM-90, MPM-373, and MPM-351 yield unprecedented data on enantiornithine tooth replacement. Although an alternating tooth replacement pattern was reported for *Archaeopteryx*^18^, previous to our study no other data on tooth cycling was known for other stem birds. Our data strongly suggest an alternating pattern of dental replacement for enantiornithines, one with z-spacing differing from two. The data presented here also show that the replacement teeth in these enantiornithines developed lingually and migrated labially at an early stage of the tooth cycle. We regard this condition as evolving independently from that seen in living crocodilians and expect that a similar pattern will be documented amongst non-avian paravian theropods. CT imaging of well-preserved avian specimens and their closest theropod relatives, like those from the Jehol Biota^47^, is likely to provide additional evidence for understanding the tooth cycle of stem birds and the deep dental homologies within archosaurs.

## Methods

MPM-90, MPM-373, and MPM-351 were scanned using a GE Phoenix Nanotom M at the Molecular Imaging Center of the University of Southern California (USC). MPM-90 and MPM-351 were scanned at 2.73 µm voxel size, 80 kV, 100 mA, exposure time 1250 ms, averaging two frames and skip one frame, 360° rotation 1000 frames, no filter. All three specimens were scanned at 10 µm voxel size, 100 kV, 200 mA, exposure time 750 ms, averaging three frames and skip one frame, 360° rotation 1440 frames, 0.1 mm Cu filter. The scans were reconstructed through GE phoenix datos| × 2 reconstruction 2.3.3.160. The three-dimensional visualization and analyses are conducted using Avizo Lite (9.2). The dentitions were also segmented and measured by tools of this software. The segmentation of MPM-90 and MPM-351 was conducted using the scans at 2.73 µm resolution.

## Supplementary Information

Supplementary Information.

## Data Availability

The enantiornithine specimens used in this study are deposited at the Museu de Paleontologia de Marília under collection number MPM-90, MPM-351, and MPM-373. Digital models of the dentitions of these enantifornithines and the µCT scan data are available at Zenodo (10.5281/zenodo.5502305).
