# Supplementary material for: Dental replacement in Mesozoic birds: evidence from newly discovered Brazilian enantiornithines
Source: Sci Rep. 2021 Sep 30;11:19349. doi: 10.1038/s41598-021-98335-8 (PMC8484441; doi:10.1038/s41598-021-98335-8)
Supplement: Supplementary file 1 — Supplementary Information. [file 41598_2021_98335_MOESM1_ESM.pdf]

**Supplementary Information for:**

## Dental replacement in Mesozoic Birds: evidence from newly discovered Brazilian enantiornithines

Yun-Hsin Wu*, Luis M. Chiappe, David J. Bottjer, William Nava, Agustín G. Martinelli

*Corresponding author: Yun-Hsin Wu
Email: yunhsinw@usc.edu

**This PDF includes:**

Supplementary figures S1
Supplementary figures S2
Supplementary figures S3
Supplementary table S1

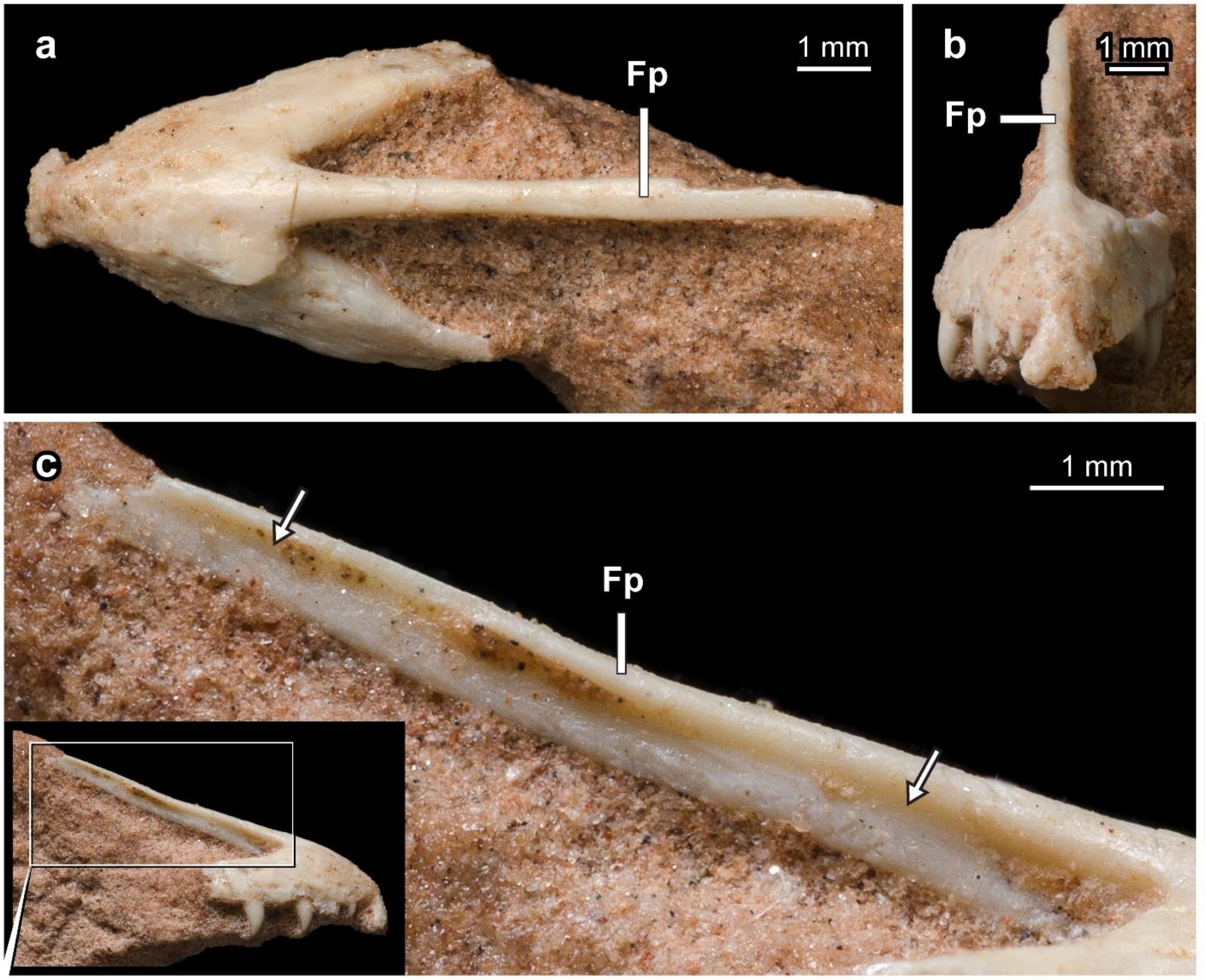


**Fig. S1. Photographs and closeup of enantiornithine specimen MPM-373.**
**a.** dorsal view; **b.** anterior view; **c.** closeup of the frontal process showing the groove (white arrows). **a.** and **b.** show the lateral compression of the specimen.
Fp: Frontal process.

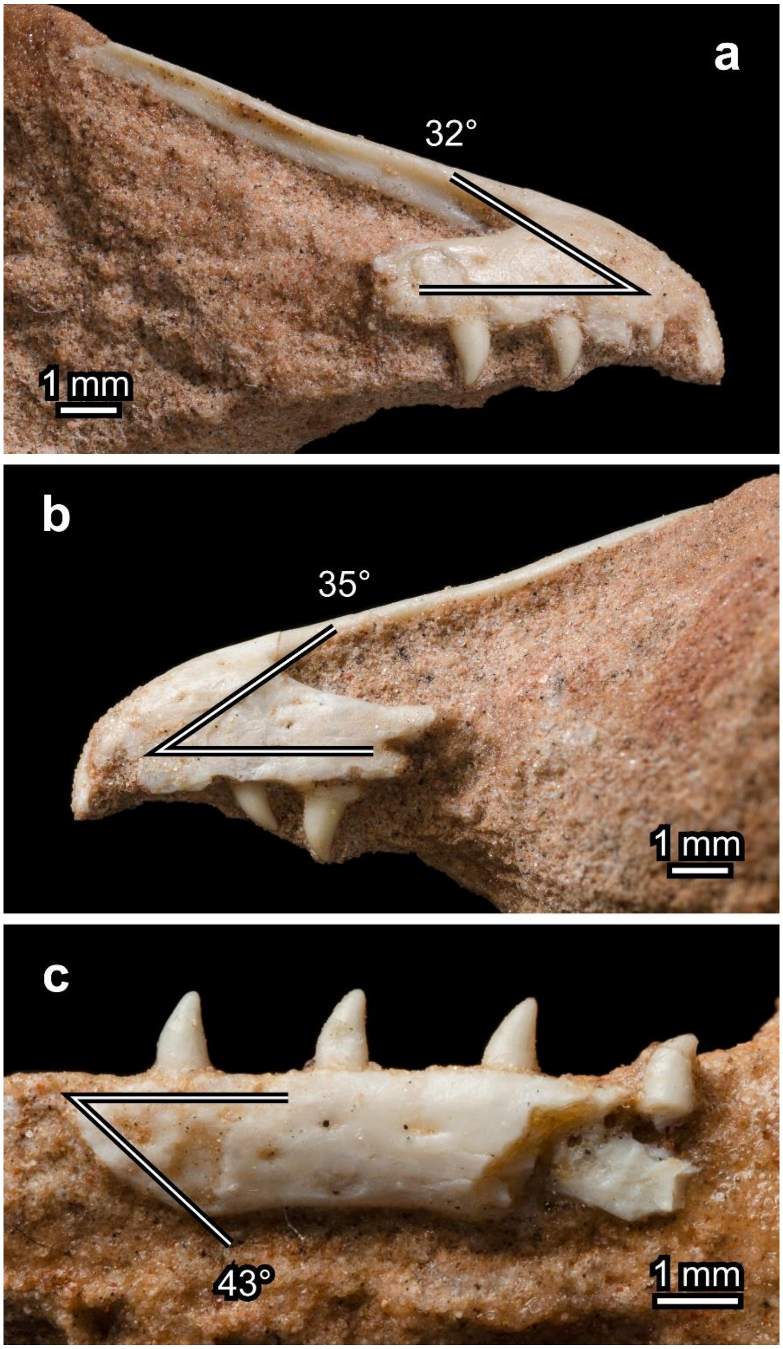


**Fig. S2. Angle measurements of MPM-373 and MPM-351.**
**a.** angle between the longitudinal axes of the frontal and maxillary processes on the right side of MPM-373; **b.** angle between the longitudinal axes of the frontal and maxillary processes on the left side of MPM-373; **c.** angle of the rostral end of MPM-351.

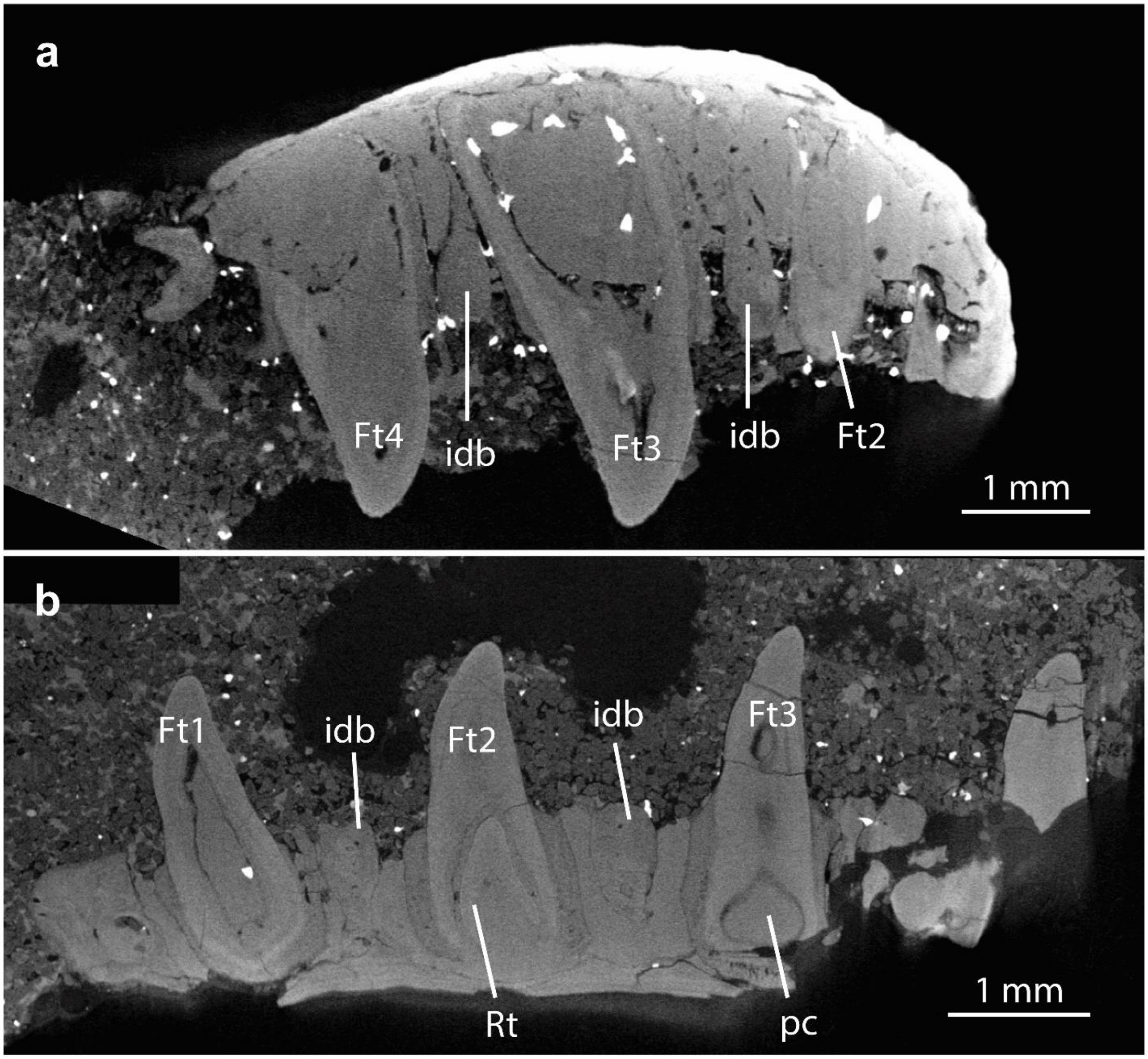


**Fig. S3. µCT images showing teeth that have grown in alveoli and porous interdental bone.**
**a.** left premaxilla of MPM-90; **b.** MPM-351 (left dentary).
Ft: functional tooth; idb: interdental bone; pc: pulp cavity; Rt: replacement tooth.

**Table S1. Prenarial length (mm) of MPM-90 and MPM-373.**
Measurements were taken through CT segmentation using Avizo Lite 9.2.

| MPM-90 | | MPM-373 | |
|---|---|---|---|
| right | 5.03 | right | 4.2 |
| left | 4.90 | left | 4.29 |
